# Sex disparities in hepatocellular carcinoma immunotherapy: hormonal and genetic influences on treatment efficacy

**DOI:** 10.3389/fimmu.2025.1607374

**Published:** 2025-05-14

**Authors:** Lei Song, Liyan Sun, Yuning Ren, Xiaodan Wang, Lei Xian

**Affiliations:** ^1^ Department of Interventional Therapy, The First Hospital of Jilin University, Changchun, China; ^2^ Pediatric Outpatient Department, The First Hospital of Jilin University, Changchun, China; ^3^ Key Laboratory of Organ Regeneration and Transplantation of Ministry of Education, The First Hospital of Jilin University, Changchun, China; ^4^ National-Local Joint Engineering Laboratory of Animal Models for Human Disease, The First Hospital of Jilin University, Changchun, China

**Keywords:** sex disparities, immunotherapy, hepatocellular carcinoma, estrogen, hormone

## Abstract

Hepatocellular carcinoma (HCC) is a highly aggressive liver cancer with a rising incidence globally. Immunotherapy, particularly immune checkpoint inhibitors (ICIs), has revolutionized HCC treatment, yet response rates remain variable. Sex-based disparities in immunotherapy efficacy have become increasingly recognized as important factors influencing treatment outcomes in HCC. This review examines the role of biological sex in HCC progression and immunotherapy responses. It discusses the epidemiology of sex differences in HCC incidence, prognosis, and therapeutic outcomes, highlighting the impact of sex hormones, such as estrogen and testosterone, on immune system function and tumor biology. Estrogen’s protective effects, including enhanced T cell activation and improved immune surveillance, contribute to better treatment responses in females, while testosterone’s immunosuppressive effects lead to poorer outcomes in males. The review also explores the influence of the tumor microenvironment, including immune cell composition and macrophage polarization, on treatment efficacy. Emerging evidence suggests that sex-specific factors, including hormonal status, should be considered in clinical trials and personalized treatment strategies. By addressing these disparities, tailored immunotherapeutic approaches could optimize efficacy and minimize toxicity in both male and female HCC patients, ultimately improving overall outcomes.

## 1 Introduction

Hepatocellular carcinoma (HCC) is the most common primary liver cancer and represents a major global health burden (1, 2). It is the sixth most prevalent cancer worldwide and the third leading cause of cancer-related deaths. The incidence of HCC has been steadily rising due to the increasing prevalence of risk factors such as chronic hepatitis B virus (HBV) and hepatitis C virus (HCV) infections, non-alcoholic fatty liver disease (NAFLD), alcohol consumption, and metabolic disorders (3–5). Despite advances in early detection and therapeutic strategies, HCC remains a highly aggressive malignancy with a poor prognosis, as it is often diagnosed at an advanced stage where curative treatment options are limited. The treatment landscape for HCC has evolved significantly over the past decade, with immunotherapy emerging as a promising approach. Immune checkpoint inhibitors (ICIs), such as programmed death-1 (PD-1) and programmed death-ligand 1 (PD-L1) inhibitors, have shown considerable potential in improving patient outcomes (6–8). In addition, other immunotherapeutic strategies, including adoptive cell therapies (e.g., chimeric antigen receptor T-cell therapy), cancer vaccines, and combination regimens with targeted therapies, are actively being explored (9–12). However, despite these advancements, immunotherapy response rates in HCC remain suboptimal, and significant inter-patient variability is observed. One underappreciated factor contributing to these differences is biological sex.

Sex disparities in cancer immunotherapy have been increasingly recognized as an important determinant of treatment efficacy and disease progression (13, 14). Differences in immune system function, hormonal regulation, genetic predisposition contribute to distinct responses between males and females (15). One of the most striking epidemiological characteristics of HCC is its significantly higher incidence in males compared to females. Global cancer statistics consistently report a male-to-female incidence ratio ranging from 2:1 to 5:1, with some geographic variations influenced by risk factor prevalence and genetic predispositions. This disparity is attributed to a complex interplay of biological, hormonal, environmental, and lifestyle factors. Several mechanisms have been proposed to explain the higher susceptibility of males to HCC. One key factor is the influence of sex hormones. Estrogen, which is more prevalent in females, has been shown to exert protective effects against HCC by modulating inflammatory pathways, reducing hepatic fibrosis, and enhancing immune surveillance (16, 17). In contrast, androgens, which are more abundant in males, are associated with increased hepatocarcinogenesis, likely due to their role in promoting cell proliferation and suppressing anti-tumor immune responses (18, 19). Beyond hormonal influences, sex-based differences in immune function also play a critical role in HCC progression and treatment responses (20, 21). Females generally exhibit stronger innate and adaptive immune responses, characterized by higher levels of pro-inflammatory cytokines, greater T-cell activation, and enhanced antigen presentation. This heightened immune activity can contribute to improved tumor surveillance and response to immunotherapy. Conversely, males tend to have a more immunosuppressive tumor microenvironment, with higher levels of myeloid-derived suppressor cells (MDSCs), regulatory T cells (Tregs), and tumor-associated macrophages (TAMs), which facilitate tumor progression and immune evasion (22, 23). These immune differences also extend to treatment outcomes. Emerging evidence suggests that female HCC patients may experience better responses to ICIs compared to males, potentially due to their stronger immune activation. However, females also have a higher incidence of immune-related adverse events (irAEs), reflecting their heightened immune sensitivity. In contrast, males often exhibit lower response rates to immunotherapy but may tolerate treatment better due to their relatively dampened immune reactivity.

This review aims to provide an updated overview of the role of sex disparities in HCC immunotherapy. We will discuss epidemiological evidence, explore underlying biological mechanisms, and evaluate the implications of these differences in clinical practice. By elucidating the impact of sex on HCC progression, immune responses, and treatment efficacy, we hope to highlight the need for sex-specific strategies in the development and application of immunotherapeutic interventions.

## 2 Epidemiology of sex differences in HCC immunotherapy

### 2.1 Sex-based variations in immunotherapy outcomes

Sex-based differences in response to immunotherapy have been increasingly recognized in HCC treatment, particularly in the efficacy of immune checkpoint inhibitors (24, 25). Checkpoint blockade therapies, such as PD-1/PD-L1 inhibitors (e.g., nivolumab, pembrolizumab, atezolizumab), have revolutionized HCC treatment, yet their effectiveness varies between male and female patients (26, 27). Female HCC patients generally exhibit better response rates to PD-1/PD-L1 inhibitors than their male counterparts. For instance, a meta-analysis of clinical trials across various cancers, including HCC, found that women had a higher overall survival benefit from anti-PD-1/PD-L1 therapy compared to men. The underlying mechanisms for these disparities may involve differential immune system activity, where females generally have stronger adaptive and innate immune responses. Additionally, estrogen has been shown to enhance T cell function and promote anti-tumor immunity, whereas testosterone may contribute to immune suppression. Beyond ICIs, sex-based variations have also been noted in other emerging immunotherapies, such as CAR-T cell therapy. Although CAR-T therapy is still in its early stages for HCC treatment, preliminary studies indicate that female patients may experience greater T cell persistence and expansion post-infusion, potentially leading to more durable responses. This finding aligns with broader observations in hematologic malignancies, where female patients have demonstrated superior CAR-T cell therapy outcomes. However, further research is needed to validate these findings in solid tumors like HCC.

### 2.2 Clinical evidence of sex disparities in HCC treatment

Sex-specific outcomes in HCC treatment have been observed in several clinical trials evaluating immunotherapy efficacy. The IMbrave150 trial, which established the combination of atezolizumab and bevacizumab as a first-line treatment for HCC, provided some insights into sex-based differences. While the trial did not specifically stratify patients by sex, subgroup analyses suggested a trend toward better outcomes in female patients receiving this combination therapy (28). Similarly, retrospective analyses of nivolumab and pembrolizumab trials indicate that female patients tend to experience longer progression-free survival and overall survival compared to males. Despite these observations, significant limitations exist in the current body of research. Most clinical trials are not explicitly designed to assess sex-based disparities, leading to potential underrepresentation and inadequate statistical power to detect meaningful differences. Moreover, hormonal status, menopausal state, and other sex-specific factors are rarely accounted for in clinical trial designs, limiting our ability to fully understand the impact of sex on treatment outcomes.

## 3 Influence of sex hormones on HCC progression and immunotherapy

### 3.1 Estrogen exerts its protective effect by enhancing anti-tumor immunity

Estrogen, a key female sex hormone, plays a significant role in modulating immune responses and influencing cancer progression (29, 30). The immunomodulatory effects of estrogen contribute to the observed sex differences in HCC incidence and response to immunotherapy. Estrogen exerts its protective effect by enhancing anti-tumor immunity. Estrogen promotes the activity of CD8^+^ T cells, which are crucial for immune-mediated tumor clearance. Estrogen enhances interferon-gamma (IFN-γ) production, a cytokine critical for T cell activation and cytotoxicity (31, 32). In contrast, lower estrogen levels in males have been associated with decreased CD8^+^ T cell function and increased immune exhaustion, which may contribute to poorer responses to ICIs. Furthermore, estrogen modulates the function of tumor-associated macrophages (TAMs) within the HCC tumor microenvironment. TAMs exist in two major phenotypes: M1, which is pro-inflammatory and anti-tumorigenic, and M2, which is immunosuppressive and promotes tumor progression (33, 34). Estrogen has been shown to shift macrophage polarization toward the M1 phenotype, thereby enhancing anti-tumor immunity (35, 36). This effect contrasts with the influence of testosterone, which promotes M2 macrophage polarization and immunosuppression.

**Figure 1:**
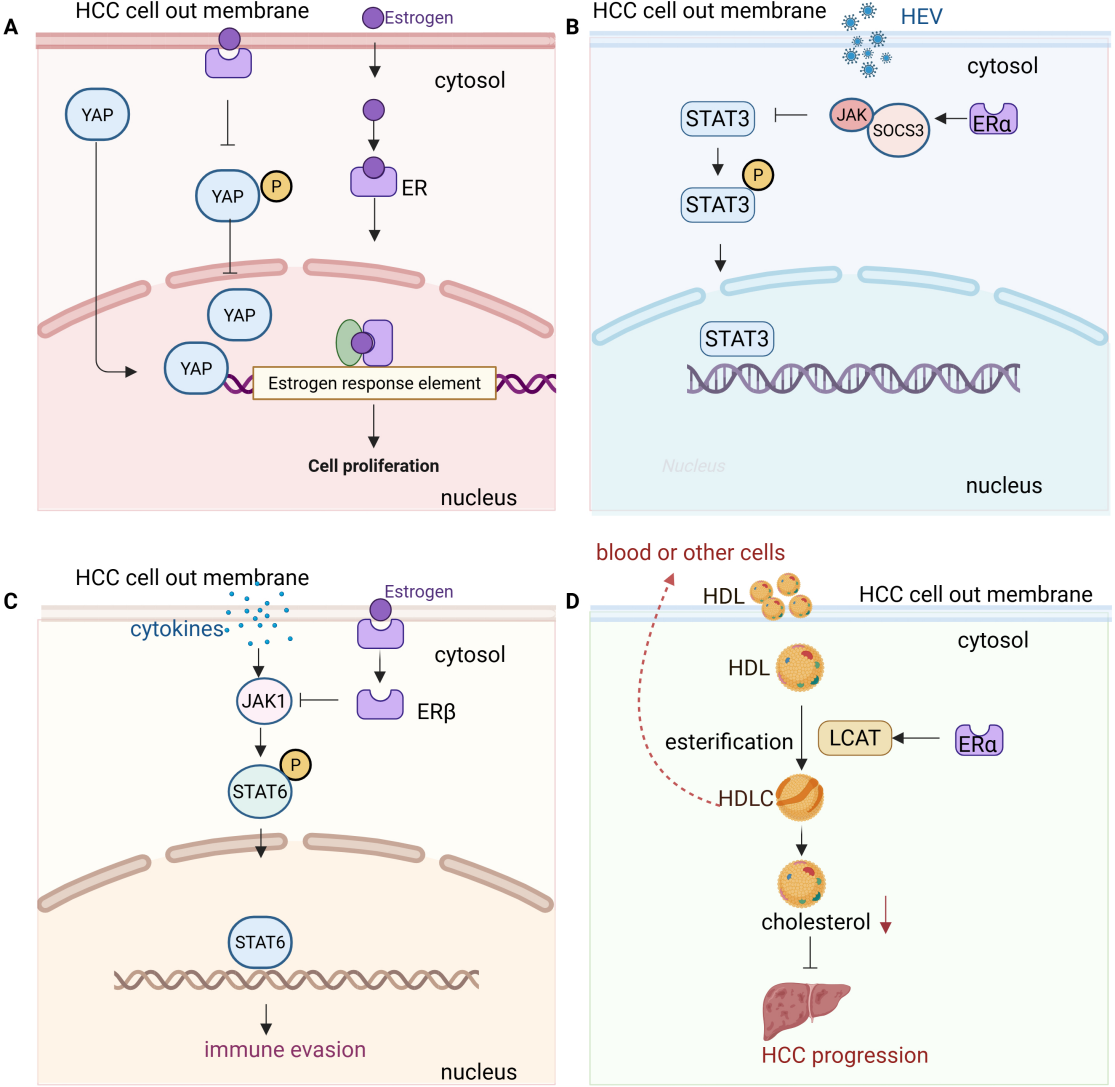
Molecular mechanisms of estrogen signaling in hepatocellular carcinoma (HCC) progression and immune modulation. **(A)** YAP signaling and ERα: Estrogen binding to ERα suppresses YAP (Yes-associated protein) signaling in HCC cells. This is achieved by enhancing YAP phosphorylation, preventing its nuclear translocation, and inhibiting downstream oncogenic pathways. The suppression of YAP by ERα contributes to tumor-suppressive effects, highlighting ERα as a potential therapeutic target in HCC. **(B)** JAK1/STAT3 signaling and ERα: Estrogen binding to ERα modulates the JAK1/STAT3 pathway in HCC cells by stabilizing suppressor of cytokine signaling 3 (SOCS3). Cytokine stimulation activates JAK1, which then phosphorylates STAT3. Phosphorylated STAT3 translocates to the nucleus to regulate target genes involved in inflammation, immune response, and tumor progression. Estrogen’s modulation of this pathway helps control the immune response and liver inflammation, contributing to reduced susceptibility to HCC. Inhibition of STAT3 activation by estrogen enhances anti-tumor immunity and inhibits HCC growth. **(C)** JAK1/STAT6 signaling and ERβ: In HCC cells, estrogen signaling through ERβ suppresses the JAK1/STAT6 pathway. Cytokine stimulation activates JAK1, which then phosphorylates STAT6, promoting its nuclear translocation and activation of target genes involved in tumor progression and immune suppression. Estrogen’s modulation of the JAK1/STAT6 axis reduces STAT6 activation, leading to a less immunosuppressive tumor microenvironment and enhancing anti-tumor immunity in HCC cells. **(D)** LCAT signaling and ERα: Estrogen activation of ERα induces the upregulation of lecithin cholesterol acyltransferase (LCAT) in HCC cells, leading to enhanced high-density lipoprotein cholesterol (HDL-C) production. This HDL-C production suppresses cholesterol biosynthesis, inhibiting tumor growth. Furthermore, HDL-C synergizes with lenvatinib, enhancing anti-tumor efficacy. Targeting the LCAT/HDL-C axis may improve immunotherapy and treatment outcomes in HCC.

Estrogen exerts protective effects in HCC through its receptors, mainly estrogen receptor alpha (ERα) and estrogen receptor beta (ERβ), which are associated with better overall survival and disease-free survival in patients (**Figure 1**). Mechanistically, ERα suppresses yes-associated protein (YAP) signaling by enhancing YAP phosphorylation, preventing its nuclear translocation, and inhibiting downstream oncogenic pathways (37). This suggests that ERα inactivation of YAP signaling contributes to its tumor-suppressive role in HCC, making it a potential prognostic marker and therapeutic target (**Figure 1A**). Additionally, estrogen influences HCC progression by modulating liver function and the immune response through ERα signaling. During HEV infection, estrogen-ERαp66 signaling regulates the STAT3 pathway, stabilizing SOCS3 and modulating the innate immune response in hepatocytes (38) (**Figure 1B**). While estrogen does not directly affect HEV replication, blocking STAT3 reduces HEV capsid protein levels, highlighting a potential link between estrogen signaling, liver inflammation, and HCC susceptibility (38). ERα activation also induces lipid accumulation and fibrosis by inducing PNPLA3 expression, which contributes to the higher prevalence and severity of fatty liver disease (FLD) in women (39). This interaction suggests that ER-α and PNPLA3 may play a crucial role in sex-based differences in FLD, potentially influencing the progression of HCC in at-risk individuals. In addition, estrogen signaling modulates ATXN7L3’s function, potentially switching HCC from a tumor-promoting to a tumor-suppressing state by facilitating deubiquitinase swapping, where ATXN7L3 and ENY2 replace USP22 (a tumor-promoting deubiquitinase) with USP27x or USP51 (potential tumor-suppressing deubiquitinases) (40). This mechanism may allow ERα to redirect ATXN7L3’s activity toward pathways that inhibit tumor progression rather than promote it (40).

Moreover, ERβ signaling can suppress pro-inflammatory cytokine production, thereby reducing chronic liver inflammation, a major risk factor for HCC. ERβ activation in macrophages has been shown to inhibit tumor growth in certain cancers by suppressing the JAK1/STAT6 pathway, which may contribute to a less immunosuppressive tumor microenvironment (41) (**Figure 1C**). ERβ plays a crucial role in CD8+ T cell-mediated anti-tumor immunity. TCR activation triggers ERβ phosphorylation, enhancing downstream TCR signaling via a non-genomic mechanism. Mutation of the ERβ phosphotyrosine switch impairs CD8+ T cell function and promotes tumor growth, while the ERβ agonist S-equol boosts TCR activation and enhances anti-PD-1 immunotherapy, suggesting ERβ as a potential target for improving cancer immunotherapy (42). Recent studies have revealed that ERβ plays a crucial role in the development of uterine corpus endometrial cancer (UCEC) by modulating the miR-765/PLP2/Notch signaling axis. This mechanism is influenced by the exosomes released by CD8+ T cells, which regulate the miR-765/PLP2 pathway, potentially limiting estrogen-driven tumor progression. Similar interactions between estrogen signaling and immune cell-derived exosomes might be present in liver cancer, suggesting a complex interplay between hormonal signaling and immune responses that could influence HCC progression (43).

G protein-coupled estrogen receptor 1 (GPER1) is a non-classical estrogen receptor that mediates rapid, non-genomic estrogen signaling (44). Unlike the classical estrogen receptors ERα (ESR1) and ERβ (ESR2), which function as nuclear transcription factors, GPER1 is a membrane-bound receptor that activates intracellular signaling cascades in response to estrogen binding. GPER1 was found to play a protective role against HCC tumorigenesis. GPER knockout in a mouse tumor model accelerated liver tumor formation, accompanied by increased immune cell infiltration, fibrosis, and elevated inflammatory factors such as IL-6 (44), activating GPER could be a potential strategy for HCC prevention and treatment. GPER1 also plays a key role in restricting macrophage proliferation in HCC. Lower GPER1 expression in macrophages correlates with increased macrophage proliferation and tumor progression, while activation of GPER1 signaling suppresses macrophage proliferation via the MEK/ERK pathway and reduces PD-L1 expression, delaying tumor growth (45). In addition to its role in macrophages, GPER1 signaling has been implicated in modulating neutrophil recruitment and NK cell cytotoxicity, which may further contribute to its protective effects against HCC (46). These findings highlight a potential therapeutic strategy targeting GPER1 to modulate the tumor microenvironment in a sex-specific manner.

Besides functions via its receptors, estrogen also exerts protective effects against HCC by upregulating lecithin cholesterol acyltransferase (LCAT), which enhances high-density lipoprotein cholesterol (HDLC) production and uptake, thereby suppressing cholesterol biosynthesis and inhibiting tumor growth through an ESR1-dependent pathway (**Figure 1D**). Additionally, HDL-C synergizes with lenvatinib to enhance anti-tumor efficacy, suggesting that targeting the LCAT/HDL-C axis could improve immunotherapy and treatment outcomes in HCC (16).

### 3.2 Testosterone suppresses immune responses in HCC

**Figure 2:**
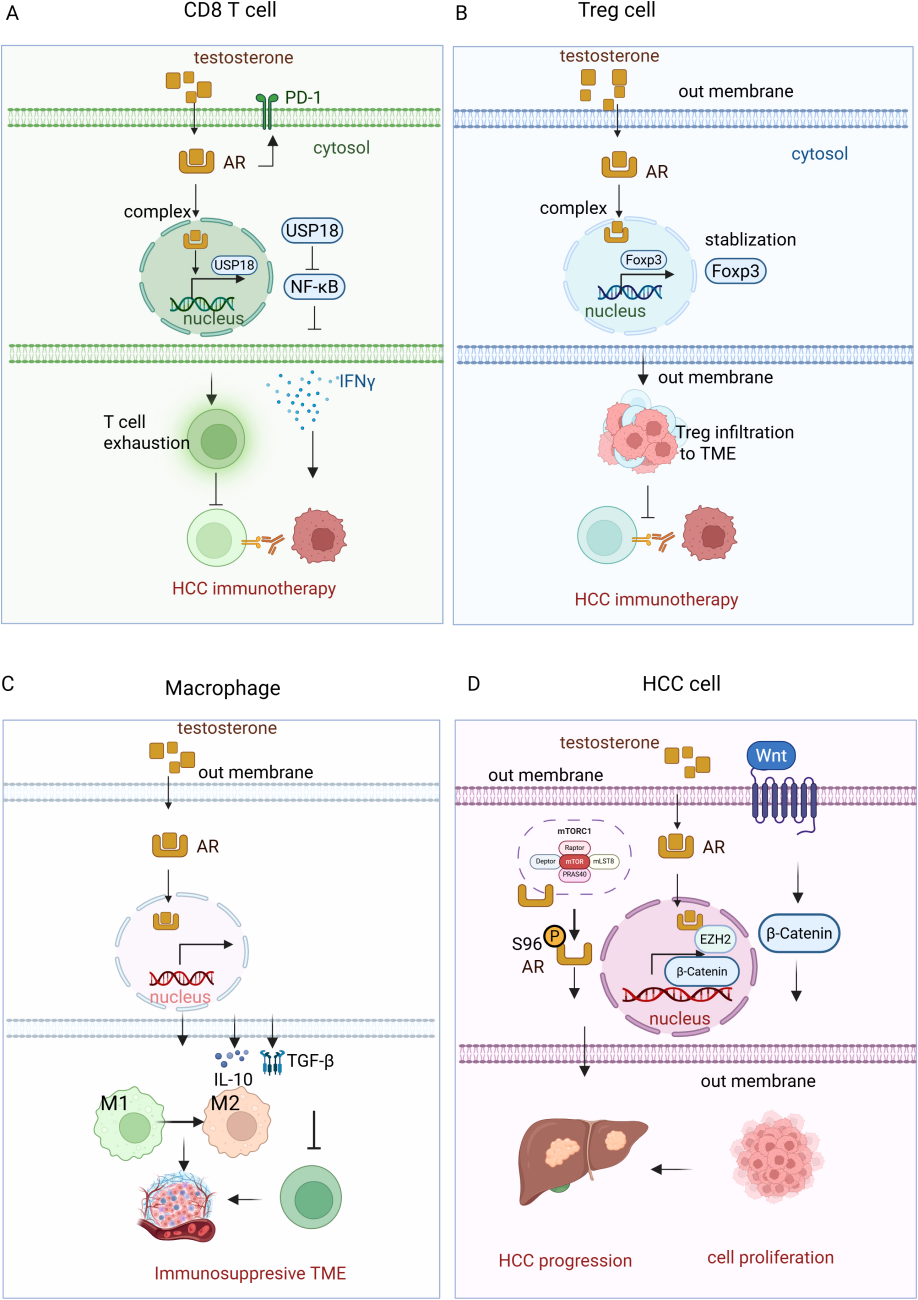
Testosterone-AR signaling pathway in HCC immunotherapy. **(A)**Testosterone binds to the androgen receptor (AR) in CD8^+^ T cells, forming a complex in the cytosol. This complex then translocates to the nucleus, where it upregulates the expression of USP18. USP18 inhibits NF-κB activity, reducing IFN-γ production. Additionally, testosterone-AR binding promotes T cell exhaustion, impairing T cell-mediated immunotherapy in HCC. **(B)** In regulatory T cells (Tregs), the testosterone-AR interaction stabilizes Foxp2, enhancing Treg cell infiltration into the tumor microenvironment (TME). This infiltration suppresses T cell immunotherapy in HCC. **(C)** In macrophages, the testosterone-AR binding increases the production of IL-10 and TGF-β, which promotes the polarization of macrophages from the M1 to the M2 phenotype. This shift suppresses cytotoxic T cell function, compromising the efficacy of immunotherapy in HCC. **(D)** In HCC cells, testosterone-AR binding upregulates the transcription of EZH2, resulting in increased levels of H3K27me3. This epigenetic modification silences Wnt signaling inhibitors, thereby activating the Wnt/β-catenin pathway, which promotes cancer cell proliferation and tumor progression. Additionally, AR interacts with mTORC1, which phosphorylates AR at S96 in response to nutrient and mitogenic stimuli. This phosphorylation enhances AR stability, nuclear localization, and transcriptional activity, further promoting lipogenesis, hepatocyte proliferation, and hepatocarcinogenesis.

In contrast to estrogen, testosterone—the primary male sex hormone—has been associated with immunosuppressive effects that may contribute to the higher incidence and poorer prognosis of HCC in males (47, 48). Testosterone has been shown to influence immune responses by modulating androgen receptor (AR) signaling, which affects various immune cells in the tumor microenvironment (**Figure 2**). Testosterone induces long-term changes in liver gene expression, including the upregulation of HCC-related genes such as Lama3 and Nox4, while suppressing immune response genes like IFNγ. These changes may contribute to the masculinized liver metabolism and immune evasion, creating an environment that favors HCC progression in males, suggesting that testosterone may play a role in promoting HCC by impairing immune surveillance and enhancing tumor-promoting pathways (49). AR mediates sex differences in cancer progression by promoting CD8^+^ T cell exhaustion in males. Specifically, AR influences the development of antigen-experienced progenitor exhausted CD8^+^ T cells in a sex-specific manner. Inhibiting the androgen-AR axis reprograms the tumor microenvironment, enhancing effector T cell differentiation and improving the response to anti-PD-1 immunotherapy, highlighting the potential for targeting androgen signaling in cancer treatment (13). Testosterone also suppresses immune responses is by downregulating the activity of CD8^+^ T cells (50). Blocking AR enhances CD8 T cell function and sensitizes tumor-bearing hosts to effective immune checkpoint blockade. This inhibition prevents T cell exhaustion, increases IFNγ expression, and improves the responsiveness to PD-1 targeted therapy, highlighting a novel mechanism of immunotherapy resistance (51). Male CD8^+^ T cells exhibit impaired effector and stem cell-like properties compared to female CD8^+^ T cells, with AR inhibiting their activity and stemness through epigenetic and transcriptional regulation (52). Androgen-axis blockade (via androgen deprivation therapy and enzalutamide) combined with anti-PD-L1 treatment synergistically restricted tumor growth in male mice (52, 53). In humans, higher AR expression in tumor-infiltrating CD8^+^ T cells correlates with T cell exhaustion, suggesting that sex-biased differences in T cell stemness contribute to cancer progression and responses to immunotherapy. Androgen receptor activation upregulates USP18, inhibiting NF-κB in antitumor T cells (54) (**Figure 2A**). Castration or abiraterone treatment enhances T-cell activity and improves the effectiveness of anti-PD-1 immunotherapy in male mice (54), suggesting that inhibiting androgen signaling could be a promising strategy to enhance immunotherapy in males.

Testosterone also exerts its effects on Tregs, which are crucial for maintaining immune tolerance but can suppress anti-tumor immunity in the context of cancer. Increased testosterone levels have been linked to the expansion of Tregs within the tumor microenvironment, contributing to immune evasion by HCC cells. This immunosuppressive effect may contribute to the lower response rates observed in male patients undergoing checkpoint inhibitor therapy. In HCC, testosterone signaling has been shown to stabilize Foxp3^+^ Tregs, enhancing their suppressive function and limiting the number of effector T cells (55) (**Figure 2B**). Additionally, androgen signaling decreases the expression of Gata2, a transcription factor for ST2^+^ Tregs, and reduces IL-33 production from airway epithelial cells in asthma models (55). These findings suggest that testosterone-induced Treg accumulation and function dampen the immune response against tumors, potentially contributing to the reduced efficacy of immunotherapies in males.

Moreover, testosterone influences macrophage polarization by promoting the M2 phenotype, which is associated with tumor progression and immune suppression. Androgens, specifically dihydrotestosterone, promote M2 macrophage polarization in allergic asthma by enhancing IL-4-induced M2 differentiation *in vitro* and *in vivo*. In mice lacking AR in macrophages, reduced M2 polarization led to decreased eosinophil recruitment and lung inflammation (56), highlighting the critical role of androgen/AR signaling in regulating macrophage polarization in diseases like asthma and prostate cancer. M2 macrophages secrete cytokines such as IL-10 and TGF-β, which inhibit the activation of cytotoxic T cells and natural killer cells, thereby reducing the overall effectiveness of immunotherapy. Testosterone-driven M2 polarization has been observed in various malignancies, including HCC, where M2 macrophages support tumor progression by creating an immunosuppressive microenvironment conducive to cancer cell survival and metastasis (**Figure 2C**).

Beside suppressing the immune response, testosterone -AR signaling also modulates HCC progression via other mechanisms. In HCC, AR upregulates EZH2 expression by binding to its promoter, thereby stimulating its transcriptional activity. This leads to increased levels of H3K27me3, which silences wingless//Int-1 (Wnt) signaling inhibitors and activates the Wnt/β-catenin pathway (57). The activation of this pathway promotes cell proliferation and tumorigenesis, correlating with tumor progression and poor prognosis in HCC patients. Furthermore, AR interacts with the mechanistic target of rapamycin complex 1 (mTORC1), which phosphorylates AR at S96 in response to nutrient and mitogenic stimuli (58). This phosphorylation enhances the stability, nuclear localization, and transcriptional activity of AR, further promoting lipogenesis, hepatocyte proliferation, and hepatocarcinogenesis. High AR S96 phosphorylation is associated with poor overall survival and disease-free survival in HCC patients, highlighting the cooperative role of AR and mTORC1 in driving liver tumor progression and providing a potential target for HCC treatment (58) (**Figure 2D**).

In conclusion, sex hormones play a critical role in shaping immune responses and influencing the outcomes of HCC immunotherapy (**Table 1**). While estrogen enhances anti-tumor immunity and improves responses to checkpoint inhibitors, testosterone promotes immune suppression and may contribute to resistance to immunotherapy. Understanding these hormonal effects is essential for developing personalized treatment strategies that account for sex-based differences in HCC immunotherapy. Targeting androgen signaling in combination with immune checkpoint inhibition holds promise for improving outcomes in male HCC patients.

**Table 1:** The role of estrogen and testosterone-regulated genes in HCC progression and immunotherapy response.

Gene name	Working mechanisms	References
Estrogen Receptor (ESR1)	Estrogen binds to ESR1, enhancing T cell activation, promoting immune surveillance, and improving treatment responses in females. ESR1 expression may serve as a predictive biomarker for immunotherapy responsiveness in female HCC patients.	(37)
Androgen Receptor (AR)	Testosterone binding to AR inhibits immune function, resulting in suppressed immune response and worse prognosis in males. Targeting AR may improve immunotherapy efficacy in male patients.	(52, 57)
CYP1A1 (Cytochrome P450)	Estrogen upregulates CYP1A1 expression, promoting the metabolism of estrogens and contributing to tumorigenesis in HCC. Altered CYP1A1 activity may influence hormone-dependent immune responses.	(59, 60)
PTEN (Phosphatase and Tensin Homolog)	Estrogen inhibits PTEN phosphorylation, enhancing tumor immune responses and inhibiting cancer cell proliferation. PTEN modulation may sensitize tumors to immune checkpoint therapy.	(61)
MMP-9 (Matrix Metalloproteinase 9)	Estrogen increases MMP-9 expression, facilitating the breakdown of extracellular matrix, aiding in tumor invasion and metastasis. MMP-9 may be associated with immune cell infiltration and tumor immune evasion.	(62)
VEGF (Vascular Endothelial Growth Factor)	Estrogen upregulates VEGF, contributing to angiogenesis and tumor growth in HCC. Anti-VEGF therapy could be tailored by sex to enhance immunotherapy outcomes.	(63)
Foxp3 (Forkhead Box P3)	Testosterone downregulates Foxp3 expression, leading to increased Treg function and immune suppression in males. Foxp3 is a potential sex-related biomarker of immune suppression.	(64)
IL-6 (Interleukin 6)	Estrogen increases IL-6 production, influencing pro-inflammatory cytokine networks and modulating immune responses in the tumor microenvironment. Sex differences in IL-6 levels may predict immunotherapy responses.	(44)
PD-1 (Programmed Cell Death-1)	Testosterone promotes PD-1 expression, leading to T cell exhaustion and immune evasion in males. Sex-specific PD-1 expression may guide ICI treatment strategies.	(54)
CTLA-4 (Cytotoxic T-Lymphocyte-Associated Protein 4)	Estrogen reduces CTLA-4 levels, enhancing T cell-mediated anti-tumor immunity in females. CTLA-4 levels may serve as a sex-influenced checkpoint biomarker.	(65)
ARID1A (AT-rich Interaction Domain 1A)	Estrogen suppresses ARID1A, contributing to chromatin remodeling, and enhancing tumor progression in HCC. ARID1A mutations have been linked to immune checkpoint blockade sensitivity.	(66)
IL-10 (Interleukin 10)	Estrogen suppresses IL-10 production in macrophages, aiding in anti-tumor immunity. IL-10 could represent a biomarker for immunosuppressive signaling in the TME.	(35)
TGF-β (Transforming Growth Factor Beta)	Testosterone upregulates TGF-β signaling, promoting immunosuppressive microenvironment and metastasis in HCC. TGF-β inhibition may reverse sex-specific immune escape.	(67)
ERBB2 (HER2/Neu)	Estrogen stimulates ERBB2 expression, promoting cancer cell proliferation and enhancing tumor survival. ERBB2 may interact with immune signaling pathways and serve as a therapeutic target.	(68)
CYP19A1 (Aromatase)	Estrogen production from CYP19A1 regulates tumor growth and immune evasion in HCC. Aromatase inhibitors may modulate sex hormone-driven immune responses.	(69)
NF-κB (Nuclear Factor Kappa B)	Estrogen modulates NF-κB activation, promoting anti-apoptotic pathways and enhancing tumor survival in HCC. NF-κB activity is linked to inflammatory immune responses and immune resistance.	(70)
AP-1 (Activator Protein-1)	Estrogen upregulates AP-1 activity, contributing to cellular proliferation and immune modulation in the tumor microenvironment. AP-1-related pathways may be differentially regulated in males and females.	(71)
BCL-2 (B-cell lymphoma 2)	Estrogen upregulates BCL-2 expression, promoting cell survival and immune evasion. BCL-2 inhibitors may synergize with immunotherapy in sex-specific contexts.	(72)
SOCS3 (Suppressor of Cytokine Signaling 3)	Testosterone promotes SOCS3 expression, suppressing immune cell function and increasing cancer cell resistance to immune therapy. SOCS3 may act as a predictive marker for immune therapy resistance in males.	(73)
PI3K/Akt Pathway Genes	Estrogen activates PI3K/Akt signaling, promoting tumor cell survival, growth, and immune evasion. PI3K inhibitors may show sex-differentiated immunomodulatory effects.	(74)
PD-L1 (Programmed Death-Ligand 1)	Estrogen upregulates PD-L1 expression in tumor cells, suppressing T cell activation and contributing to immune escape. PD-L1 expression levels may differ by sex and influence checkpoint inhibitor efficacy.	(74)
Fas (CD95)	Testosterone regulates Fas expression, influencing apoptosis resistance in cancer cells and immune cells. Fas-mediated cell death pathways may show sex-dependent regulation in HCC.	(75)
STAT3 (Signal Transducer and Activator of Transcription 3)	Estrogen activates STAT3 signaling, promoting tumor progression and immune evasion in HCC. STAT3 inhibition may have sex-specific immunotherapeutic effects.	(70)
JAK2 (Janus Kinase 2)	Testosterone activates JAK2/STAT signaling, promoting tumor growth and immune suppression. Targeting JAK2 could counter male-biased immune resistance in HCC.	(76)
IL-2 (Interleukin 2)	Estrogen promotes IL-2 production, which enhances T cell proliferation and tumor immunity in females. IL-2 signaling may be more responsive in female patients receiving immunotherapy.	(77)
GR (Glucocorticoid Receptor)	Estrogen and testosterone modulate GR signaling, influencing immune responses and tumor progression. GR may serve as a sex-linked immunomodulatory node.	(78)

## 4 Genetic and molecular differences in immune response between males and females

The immune response is significantly influenced by genetic and molecular factors, and gender-based differences in immune regulation play a crucial role in disease outcomes, particularly in cancers (14, 79). There are substantial differences between males and females in the composition and function of both innate and adaptive immune systems. These differences are rooted in the distinct genetic makeup between the sexes, especially the X and Y chromosomes, which harbor genes involved in immune function. In this section, we will explore how X and Y chromosome-linked immune regulatory genes influence immune responses in HCC, and how sex-based variations in immune checkpoints and cytokine signaling may affect treatment outcomes.

### 4.1 X chromosome-linked immune regulatory genes

The X chromosome is unique because females have two copies of it, while males have only one. This genetic difference results in a differential expression of genes linked to the immune system. Many immune-related genes reside on the X chromosome, and their expression can have significant effects on the immune system. Key genes such as FOXP3, TLR7/8, and others are of particular interest in understanding the gender differences in immune responses.

#### 4.1.1 FOXP3

This gene is crucial for the development and function of Tregs, which play a pivotal role in maintaining immune tolerance and preventing autoimmune reactions (34, 80, 81). The expression of FOXP3 is sex-biased, with higher levels of FOXP3 expression in females than males. This is largely due to the presence of two X chromosomes in females, leading to a higher dose of immune regulation. In HCC, the regulatory role of Tregs, influenced by FOXP3, can contribute to tumor progression by suppressing effective immune responses against cancer cells. High expression of FOXP3 in HCC cells, particularly the Δ3,4-FOXP3 splice variant, is associated with better survival, reduced recurrence, and early-stage disease. FOXP3 suppresses HCC cell proliferation and invasion, likely through the TGF-β/Smad2/3 signaling pathway, although the Δ3,4-FOXP3 variant shows reduced tumor-inhibiting effects (64). The sex-based differences in FOXP3 expression may therefore influence the progression of HCC and the efficacy of immunotherapies targeting Tregs.

#### 4.1.2 Toll-like receptor 7 and Toll-like receptor 8

These genes, located on the X chromosome, are essential for recognizing pathogen-associated molecular patterns (PAMPs) and triggering innate immune responses. TLR7, in particular, has been implicated in the activation of type I interferons and the promotion of anti-tumor immunity. TLR7 evades X chromosome inactivation, leading to biallelic expression in immune cells such as B lymphocytes, which enhances immune responses and contributes to the increased susceptibility to systemic lupus erythematosus (SLE), as well as potentially impacting the efficacy of immune therapies targeting TLR7 (82). Males, with a single copy of the X chromosome, exhibit a reduced TLR7 expression compared to females, who have two copies. This difference in TLR7 expression leads to a heightened response to viral infections and may also influence the immune response to HCC, where immune surveillance and the inflammatory microenvironment play critical roles. The TLR7 agonist Imiquimod inhibits HCC by suppressing the self-renewal of cancer stem cells through the TLR7-IKK-NF-κB-IL6 signaling pathway (83). This repression reduces cell proliferation, mammosphere formation, and stem cell numbers, suggesting its potential as a therapeutic approach for HCC. Moreover, TLR7 signaling has been associated with the activation of dendritic cells and the subsequent initiation of adaptive immunity, both of which are crucial for an effective anti-tumor response (84).

#### 4.1.3 Other X-linked immune genes

Beyond FOXP3 and TLR7/8, several other immune regulatory genes reside on the X chromosome. For example, genes involved in the development of immune cells, such as CD40L and the signal transducer IRF5, are also located on the X chromosome (85). The differential expression of these genes between males and females contributes to sex differences in immune responses. For instance, females tend to have a more robust immune response to infections and vaccines, a characteristic that may also affect their ability to mount an immune response against cancer cells, including those in HCC.

#### 4.1.4 Sex-based differences in T cell and innate immune responses

T cell responses, including cytotoxic T cell (CD8^+^) activity, are crucial for the immune system’s ability to detect and kill tumor cells (86). In addition to Tregs, the activation of CD8^+^ T cells is essential for the anti-tumor immunity in HCC. Females often exhibit stronger CD8^+^ T cell responses compared to males (87). This is partially due to differences in the X-linked immune genes, such as IL-7R, which is involved in T cell survival and differentiation. Furthermore, invariant natural killer T (iNKT) cells, a subset of innate immune cells that bridge innate and adaptive immunity, exhibit sex differences in their activation and function. These differences may contribute to the more effective immune surveillance observed in females. In addition, the innate immune response, including the activity of macrophages, neutrophils, and dendritic cells, also differs between sexes. Females tend to have stronger innate immune responses, which can influence the tumor microenvironment (TME) in HCC (88). These cells are crucial for recognizing tumor cells and initiating the adaptive immune response, and sex-based variations in their function may play a role in HCC progression.

### 4.2 Y Chromosome contributions to immune modulation

The Y chromosome is another key player in sex-based immune differences. Unlike the X chromosome, which contains many immune-related genes, the Y chromosome has fewer immune-regulatory genes (89). However, some of these Y-linked genes, particularly those related to male sex determination and spermatogenesis, may also influence immune function.

#### 4.2.1 Sex-determining region Y and immune function

The SRY gene, located on the Y chromosome, is essential for male sex determination and the development of testes (90). SRY was found to be overexpressed in approximately 84% of male HCC patients, suggesting its involvement in male hepatocarcinogenesis (91). Both male and female liver-specific transgenic (TG) mice exhibited accelerated DEN-induced hepatocarcinogenesis compared to wild-type (WT) controls. The mechanism underlying this enhanced tumorigenesis involved increased liver injury, inflammation, fibrosis, and hepatocyte proliferation, driven by the activation of Sox9 and platelet-derived growth factor receptor α (PDGFRα)/PI3K/Akt and c-myc/CyclinD1 signaling pathways. SRY (through its downstream factor SOX9) impairs the differentiation of liver progenitor cells into hepatocytes, contributing to chronic liver inflammation and fibrosis, thereby promoting hepatocellular carcinoma (HCC) progression (92). These findings suggest that SRY and its downstream targets play a critical role in male-specific HCC development, offering insights into gender disparities in liver cancer and potential sex-specific therapeutic strategies (93). Beyond its role in sexual differentiation, SRY has been shown to influence immune responses. SRY may contribute to sex-based differences in immune responses by modulating T cell activation, promoting the expansion of Tregs, and influencing TAM polarization toward an immunosuppressive phenotype (94). Additionally, SRY, via its downstream target SOX9, promotes hepatocellular carcinoma (HCC) progression by upregulating CXCL5 expression and activating the CXCL5/CXCR2 signaling axis, thereby enhancing tumor cell proliferation and invasion. This axis also stimulates PI3K-AKT and ERK1/2 pathways and promotes neutrophil and macrophage infiltration, contributing to a pro-tumorigenic microenvironment (95). The interplay between SRY and AR signaling may further contribute to immune evasion mechanisms, potentially explaining the higher incidence and poorer prognosis of HCC in males (96). Understanding the immunoregulatory role of SRY provides valuable insights into the sex-specific tumor microenvironment and its impact on HCC progression.

#### 4.2.2 Other Y-linked genes

Other genes on the Y chromosome, such as ubiquitously transcribed tetratricopeptide repeat gene on the Y chromosome (UTY), may also contribute to immune regulation. While the exact mechanisms are still being studied, it is believed that these genes play a role in modulating the immune system’s response to tumors, including HCC.

#### 4.2.3 Differences in immune regulatory pathways between males and females

Male and female immune systems differ not only at the genetic level but also in how immune regulatory pathways are activated. In males, the presence of the Y chromosome may lead to differences in the expression of cytokine receptors, cell surface markers, and immune checkpoint molecules (97, 98). These differences contribute to a distinct immune response to tumors in males and females. In particular, males may exhibit less robust immune surveillance due to differences in immune checkpoint regulation, which is crucial for controlling immune responses in HCC.

## 5 Conclusions and future directions

Sex-based differences in immune responses represent a crucial factor in determining the efficacy of immunotherapies, including those used to treat HCC (99, 100). These differences arise from genetic, hormonal, and immunological factors that vary between males and females, and they significantly affect both innate and adaptive immune responses. However, it is important to note that much of the existing evidence regarding hormone-immune interactions is derived from preclinical studies or retrospective clinical observations. Direct causal links between sex hormones and differential immunotherapy outcomes remain to be established in prospective, well-controlled human studies. As such, the mechanisms proposed herein should be interpreted with caution and considered as hypotheses requiring further validation. Genetic variations linked to the X and Y chromosomes, immune checkpoints, and cytokine signaling pathways further compound these differences. As such, a deeper understanding of the underlying mechanisms behind these disparities could provide a foundation for the development of more effective and personalized immunotherapeutic approaches. Current immunotherapies, such as immune checkpoint inhibitors, have demonstrated varying success across genders. Female patients, for instance, may experience better responses to therapies targeting PD-1/PD-L1, owing to the typically higher expression of immune checkpoints in females. On the other hand, male patients may benefit from alternative therapeutic strategies that better address their distinct immune response profiles, such as those modulating the Y chromosome-linked genes or adjusting the inflammatory microenvironment. Understanding how these sex-based differences influence treatment response is a key step toward improving the efficacy of immunotherapy in HCC.

One promising direction involves the development of personalized treatment strategies that take into account the unique immune landscapes in males and females. Specifically, patient stratification based on circulating sex hormone levels (e.g., estrogen, progesterone, testosterone) may help optimize immunotherapeutic response and minimize immune-related adverse effects. Integrating hormonal modulation with immune checkpoint inhibitors could potentially enhance the therapeutic response, especially in females, who often exhibit stronger immune responses due to estrogen-related pathways. Similarly, modulating testosterone in males could help balance immune responses without triggering excessive inflammation. Emerging preclinical data suggest that co-targeting hormone receptors (e.g., estrogen receptor α or androgen receptor) alongside immunotherapy could enhance anti-tumor immunity. This opens a translational window to combine hormone therapy with immune checkpoint inhibitors, especially in sex-biased cancers such as HCC. These strategies would mark a significant shift from the current one-size-fits-all approach, offering more tailored, sex-specific treatments for HCC and other cancers. To better incorporate sex as a biological variable in immunotherapy development, we propose that future clinical trials adopt sex-stratified designs, where patients are grouped by sex and hormonal status to uncover differences in efficacy and toxicity. Adaptive trial frameworks with interim sex-based analyses could enable dynamic treatment adjustments. Moreover, the integration of hormonal co-therapies—such as aromatase inhibitors or anti-androgens—alongside immunotherapy warrants systematic evaluation. Regulatory agencies should also encourage early-phase trials to include sex-specific analyses to inform biomarker validation and therapeutic decisions. Despite the clear potential of sex-informed immunotherapy, several challenges remain, including the need to identify reliable sex-specific biomarkers, understand sex-related differences in the TME, clarify the influence of hormonal fluctuations on treatment outcomes, and develop immunomodulators that target sex-specific pathways. Addressing these gaps will be essential for translating mechanistic insights into effective, personalized cancer immunotherapies.

While the promise of sex-based immunotherapy is clear, several key challenges remain that must be addressed in future research to realize its full potential (1). Sex-stratified clinical trials: Most trials don’t separate data by sex, obscuring gender-specific effects. Large-scale trials considering sex, age, and hormonal status are needed (2). Sex-Specific Biomarkers: Identifying biomarkers for sex-related immune response differences is crucial for personalized treatment (3). TME variations: Differences in the TME between sexes affect immune responses and treatment outcomes, requiring further investigation (4). Hormonal influence: The role of sex hormones in immune regulation and therapy efficacy, especially during hormonal fluctuations, needs further study (5). Sex-specific Immunomodulators: Developing therapies targeting sex-specific immune pathways and modulating sex hormones could improve treatment outcomes.

In addition, future research should prioritize the identification and validation of sex-specific immunotherapy biomarkers. Notable candidates include FOXP3, which is involved in regulatory T cell function and has shown differential expression patterns between sexes; PD-L1, whose expression levels vary based on hormonal influence; ESR1 and AR, which mediate estrogen and androgen signaling respectively and impact immune surveillance; and XIST, a non-coding RNA regulating X-chromosome inactivation that may influence immune-related gene expression in females. Exploring these biomarkers in the context of clinical outcomes could pave the way for sex-informed treatment stratification and improved therapeutic efficacy.
